# Research evidence and implementation gaps in the engagement of people with lived experience in mental health and substance use research: a scoping review

**DOI:** 10.1186/s40900-023-00442-5

**Published:** 2023-05-11

**Authors:** Lisa D. Hawke, Natasha Y. Sheikhan, Sara Roberts, Shelby McKee

**Affiliations:** 1 Centre for Addiction and Mental Health, Toronto, ON Canada; 2 University of Toronto, Toronto, ON Canada

**Keywords:** Patient engagement, Patient and public involvement, Patient-oriented research, Lived experience, Evidence gaps, Research gaps

## Abstract

**Background:**

There is growing recognition that engaging people with lived experience (PWLE) in mental health and substance use research improves the quality of the research in terms of relevance to the population and the feasibility of the work. Engagement also provides positive opportunities for research teams and the PWLE engaged. However, there are many gaps in the research on PWLE engagement. This scoping review synthesizes the gaps in the implementation of PWLE engagement and in the research on engagement as presented by research teams engaging PWLE in their work.

**Method:**

A systematic electronic database search was conducted in 2022 for published articles on PWLE engagement in mental health and substance use research. Potential articles were screened for relevance. The search led to 49 final articles included in the review. The 49 articles were then coded using codebook thematic analysis to answer two research questions: (1) What are the research evidence gaps regarding the engagement of PWLE in mental health and substance use research?; and (2) What are the gaps in implementing PWLE engagement in mental health and substance use research? PWLE were engaged in the conduct of this review.

**Results:**

Results showed that research evidence gaps include further work on conceptualizing engagement; developing resources, tools, and practice recommendations to support research teams; increasing diversity in evaluations of engagement; and evaluating engagement, including its impact on the research, on PWLE, and on researchers. Implementation gaps included several broader institutional gaps and gaps in the day-to-day practice of engagement.

**Conclusions:**

Despite progress in PWLE engagement in mental health and substance use research in recent years, research evidence and implementation gaps remain. Research teams are encouraged to consider these gaps and conduct research and implementation activities to address them in a rigorous manner.

## Abstract (summary)

There is growing recognition that engaging people with lived experience (PWLE) in mental health and substance use research improves the quality of the research by making it more relevant to the population and more feasible. Engagement also provides positive opportunities for those working in this way. However, many questions remain unanswered in terms of PWLE engagement. We reviewed the published literature to identify gaps in the research on PWLE engagement and on the ways PWLE engagement is practiced in mental health and substance use research. We identified 49 articles addressing these issues. We examined each article to identify (1) research evidence gaps regarding the engagement of PWLE in mental health and substance use research; and (2) gaps in the way PWLE engagement is put into place in mental health and substance use research. We found that research evidence gaps include the need for further research work to understand what engagement and lived experience are; the need to develop resources, tools, and practice recommendations to support PWLE engagement; the need to increase the diversity of the PWLE engaged; and the need to evaluate the impact of engagement on the research, on PWLE, and on researchers. Gaps in the practice of PWLE engagement included several broader institutional gaps and gaps in the day-to-day practice. Despite progress in PWLE engagement in research in recent years, many gaps remain. Research teams are encouraged to conduct research to clarify these aspects of engagement and to implement engagement in ways that address these gaps.

## Background

There is an ongoing movement toward engaging people with lived experience (PWLE) of mental health and substance use challenges in research about them and their needs [1]. Moving beyond considering PWLE as research participants, patient-engaged or patient-oriented research practices, i.e., research that includes PWLE, call for their involvement directly in research processes, as advisors, co-researchers, full partners, or in various other research-related roles. In engagement roles, PWLE can advise on, collaborate on, co-design, and/or lead many aspects of research, such as setting research priorities, establishing methodologies, conducting research, analyzing and interpreting data, and conducting knowledge translation activities [2]. PWLE can be engaged across study designs and research topics [2–4]. The engagement of PWLE can be seen as an ethical imperative and anti-oppressive practice in the context of inequities that have occurred in healthcare research and clinical practice [5], and indeed emerged from disability rights and consumer/survivor movements [6]. In patient-oriented research, which is strongly rooted in pragmatism, research evidence and experiential knowledge are equally valued [7].

It should be noted that terminology to refer to PWLE in this sphere is varied, including such terms as ‘patient’, ‘service user,’ ‘consumer,’ and ‘people with lived experience,’ among others [4]. ‘Patient’ is one of the most commonly used identifiers in the literature, used in terms such as ‘patient and public involvement,’ and ‘patient engagement.’ However, the stigma associated with mental illness should be kept in mind when choosing terminology. PWLE involved in our engagement activities, including the current review, have expressed that the term ‘patient’ does not reflect the role they bring to projects as mental health experiential experts and research advisors [8]. We, therefore, use the term PWLE herein. It should also be noted that while most literature discussing the engagement of PWLE refers to lay people without academic expertise in mental health and substance use, some also include PWLE who are academic mental health researchers themselves, who contribute both lived experience and academic insights to their work [9].

A number of reviews have been conducted on the engagement of PWLE in mental health and substance use research [2, 4, 10, 11]. These show that research engaging PWLE has increased substantially in recent years, in a fast-paced research climate in which engagement has emerged as a growing priority. A recent PWLE-engaged review of the impacts of engagement suggests that engaging PWLE in research can bring many benefits to the research itself, as well as to the individuals engaged and to the researchers working in this manner [4]. The literature suggests that when PWLE and families are engaged in mental health and substance use research, the resulting research is more likely to be aligned with the needs and priorities of the target population, becoming more likely to be feasible, easily adopted, implemented, and sustainable. However, these outcomes have been derived largely from qualitative studies, commentaries, and descriptive pieces highlighting the experiences of those engaged, which has been expressed as a limitation.

Barriers, facilitators, and best practices in engaging PWLE in health research, including mental health and substance use research, have also been identified [4, 12, 13]. Facilitators exist at the level of the individuals engaged and the researchers, guiding research teams. Facilitators point to best practices in engagement, such as engaging PWLE early in the research process, providing a safe and supportive environment for PWLE, ensuring clear communication and roles, demonstrating flexibility in engagement processes, and embedding engagement in the institutional culture. In doing so, it is important to attend to the barriers to effective engagement, such as avoiding tokenistic engagement, carefully managing conflicting views and negative perceptions of engagement, addressing stigma, and navigating funding constraints that sometimes limit the breadth, scope, and timeline of engagement activities.

Despite the growing amount of guidance provided by the literature, the evidence base supporting PWLE engagement in mental health and substance use research remains limited. Research engaging PWLE is required to clarify various aspects of PWLE engagement. Research teams who are conducting mental health and substance use research that engages PWLE and families are ideally positioned to identify the research evidence and implementation gaps. As they navigate engagement activities, it is critical to identify what additional information would support them in their engagement practices, and where they are encountering the greatest research evidence gaps.

This scoping review synthesizes the research evidence and implementation gaps on the engagement of PWLE in mental health and substance use research as expressed by research teams engaging PWLE in their research. PWLE were engaged in the conduct of this review to increase relevance.

## Methods

This review is a secondary analysis of a companion scoping review on barriers, facilitators, and impacts of PWLE engagement [4]. The review was conducted following the PRISMA Extension for Scoping Reviews (PRISMA-ScR) guidelines [14]. Lived experience engagement was conducted within this review and is reported on using the Guidance for Reporting Involvement of Patients and the Public (GRIPP2) checklist for reporting patient and public involvement [15]. GRIPP2 results are reported in Table 1. We selected the scoping review methodology to conduct a broad investigation of research evidence and implementation gaps identified in the extensive literature on the topic of engagement [16]. A formal scoping review protocol was not published.

**Table 1:** Guidance for Reporting Involvement of Patients and the Public (GRIPP2) reporting checklist for lived experience engagement in research

Section and topic	Description
1: Aim	People with lived experience were engaged in this study in order to enhance the relevance of the issues reviewed, as well as the research process, interpretations, and reporting.
2: Methods	Multiple members of the project team are PWLE, with a range of levels of experience in mental health and substance use research. The title and abstract screening process and the full-text screening process both included team members who are PWLE. The project was presented at a lived experience advisory committee at the Centre for Addiction and Mental Health, where the discussion included the use of language, overall agreement with the identified gaps, and the importance of continuing the line of work to address the gaps. In addition, the project was presented at a unit meeting of 8 team members for discussion, in which multiple team members were PWLE and had a range of research experience, from junior to senior roles. Feedback from all sources of PWLE was incorporated in all stages of the review.
3: Study results	The engagement of PWLE, including non-academic and academic contributors, ensured that a rigorous understanding of PWLE engagement was brought to all study stages. PWLE co-generated and reviewed the research questions and findings. They agreed with the findings and emphasized the importance of conducting future research to address the gaps.
4: Discussion and conclusions	Members of the research team were PWLE, and additional lay PWLE insights were sought through a PWLE advisory committee meeting, which ensured that PWLE perspectives were embedded through the review; there were no substantial challenges or negative effects of engagement in the conduct of this review.
5: Reflections/ critical perspective	The engagement of PWLE was a core component of this work, which emerged from a research unit specializing in lived experience research. The engagement process is a valuable investment of time and resources that strengthens confidence in the reporting of all research projects in the unit and is particularly important to research focused on PWLE engagement.

### Research question

Among research teams engaging PWLE in their mental health and substance use research, what are the identified research evidence and implementation gaps regarding PWLE engagement? Research evidence gaps are areas in which future research is required to advance the science of PWLE engagement. Implementation gaps are areas in which greater clarity around the implementation of PWLE engagement practices is required to improve engagement activities. The PCC framework [17] (population, concept, context) was used to refine the research question and identify relevant studies. This review addresses literature on PWLE (population), engaged in mental health and substance use research (concept), in studies conducted in research settings (context). Articles that involved research conducted outside of this field were excluded.

### Identifying relevant studies

A systematic electronic database search was conducted in June 2022 for articles published from 2011 to 2022, in Medline (Ovid), CINAHL (EBSCO), and PsycINFO (ProQuest). As the concept of engagement does not have consistent terminology, pilot searches were conducted to identify the most relevant keywords. Final keywords led to a search strategy including “patient engag*” or “patient involv*” or “patient participat*” or “youth engag*”. The research component was captured using search terms such as co-researcher*, co-investigat*, consult*, advis*, team*, “expert* by experience”, and “patient* as partner*”. A range of mental health search terms was also used, including “mental health”, “mental illness*”, “mental disorder*”, psychiatr*, “substance use”, “substance-use”, “mental distress” and “psycholog* distress”, to limit the findings to engagement conducted within the mental health and substance use sphere. “Impact,” “impact*” and “outcome*” were also included as search terms to identify papers with concrete findings from research studies. This core set of search terms was optimized for each database. A sample of the search strategy as conducted in the Medline database is provided in Table 2. The search was originally conducted for a companion publication focusing on the impacts of engagement [4]. The resulting articles were re-analyzed to answer the current research question, with a date limitation of 2017–2022 (past five years) to provide up-to-date guidance on current research evidence and implementation gaps.

**Table 2:** APA Psycinfo sample search strategy

Order	Search term
1	Patient Participation/
2	((patient* or client* or public or “service user*” or youth or consumer* or citizen*) adj2 (participat* or engag* or invol*)).mp.
3	Exp mental health/
4	Mental health.mp.
5	Exp Mental Disorders/
6	Mental disorder*.mp.
7	((mental* or psychiatr* or psycholog*) adj2 (health* or ill* or hygiene or disorder* or distress*)).mp.
8	((drug* or substance* or alcohol*) adj2 (abus* or addict* or depend* or misus* or use* or dependen* or disorder*)).mp.
9	((improv* or strength* or inform* or increase* or impact* or facilitat* or support*) adj3 (research* or method* or design or outcome* or recruit* or study or team*)).tw.
10	(“liv* expertise” or “peer* researcher*” or “co-researcher*” or “expert* by experience*” or “patient* partner*” or “patient* advisor*” or “co-produc*” or “co-design”).mp.
11	1 or 2 or 10
12	3 or 4 or 5 or 6 or 7 or 8
13	9
14	11 and 12 and 13
15	Limit 14 to yr = 2012-Current

Adj is an adjacency operator that searches for a term within *n* words of another term; mp is a multi-purpose search that queries popular fields such as title, abstract, author-supplied keyword, and heading word; tw is a field code that searches for terms within the title, abstract, and key concepts

### Screening and selecting studies

**Fig. 1:**
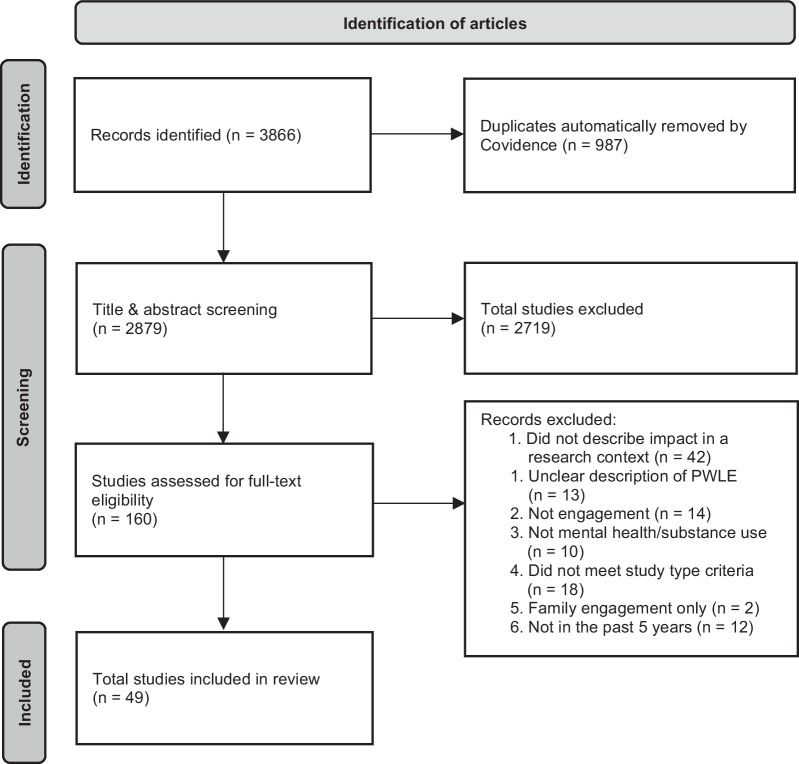
PRISMA flow chart for articles identified in the scoping review

The articles resulting from the database search were uploaded into the Covidence systematic review software [18], where duplicates were automatically removed. Three reviewers (of whom two had lived experience) screened titles and abstracts based on the eligibility criteria (Table 3). Two reviewers then conducted the full-text review to identify the final article set. The results of the screening process are illustrated in the PRISMA diagram (Fig. 1).

**Table 3:** Eligibility criteria

Eligibility criteria	Exclusion criteria
Research describing a study engaging PWLE of mental health or substance use	Does not describe a study
Discusses the engagement of PWLE of mental health or substance use challenges	Defines engagement as treatment retention or engagement in clinical service decisions, not research
Published between 2017 and 2022	Published before 2017
Published in English	Focuses primarily on neurological, developmental, or physical disorders
	Reviews, protocols, conference abstracts
	Focuses only on family engagement

*Note: PWLE* people with lived experience

### Charting and coding the data

Within the selected articles, the data were summarized using codebook thematic analysis in NVivo 12 software [19]. Codebook thematic analysis is a pragmatic approach that combines the inductive development of codes and themes/categories, which are then used deductively to code the remaining data, with constant openness to new codes and themes [20]. Codebook thematic analysis was a pragmatic choice and was used to identify key topics related to the scoping review’s main concepts. Codes and themes were therefore sought in two broad a priori categories: (1) research evidence gaps to be addressed in future research, and (2) implementation gaps to be addressed in the practice of engagement. Study objectives were also coded. In NVivo, line-by-line coding of the text of the manuscripts was conducted by the first author for the first 33% of the articles. This inductive stage led to the creation of a codebook, combing nodes and subnodes describing the data extracted from the articles. A second coder used the codebook to code the remaining articles. Bi-weekly meetings of two to four authors were held to discuss the coding process and deliberate on any newly emerging codes. Basic study descriptive information was extracted into an Excel spreadsheet.

### Synthesizing the data and reporting

Data were narratively summarized based on the categories and themes that were extracted from the included manuscripts.

### Reflexivity

This project was carried out by a research team with experience in and a commitment to engaging PWLE in mental health and substance use research. Members of the team have previously published on engagement and several of their articles were included in the final search. While this gave them the background knowledge needed to conduct this work, they have also reflected on their openness to engagement PWLE in research and the positive views that may have influenced this review. The team discussed their potential biases and opinions on an ongoing basis to maintain a stance of reflexivity in the work.

## Results

The characteristics of the 49 included articles are provided in Table 4. The largest proportion of articles emerged from the United Kingdom and Canada and had the objective of describing or discussing engagement activities. Among the 49 articles reviewed, overarching themes were extracted in two main categories of research questions: (1) the research evidence gaps regarding the engagement of PWLE in mental health and substance use research; and (2) the implementation gaps in engaging PWLE in mental health and substance use research. The majority of papers focused on PWLE primarily with mental health challenges, with only three specific to the substance use disorder sphere [21–23]. An overview is provided in Table 5.

**Table 4:** Overview of studies included

Characteristics		n (%)	References
Country	United Kingdom	18 (36.7%)	[22–39]
	Canada	10 (20.4%)	[21, 40–48]
	Australia	7 (14.3%)	[49–55]
	Australia and New Zealand	6 (12.2%)	[56–61]
	United States	3 (6.1%)	[62–64]
	Norway	2 (4.1%)	[65, 66]
	Ireland	1 (2.0%)	[67]
	Germany	1 (2.0%)	[68]
	Sweden	1 (2.0%)	[69]
Year of Publication	2021–2022	16 (32.7%)	[25, 26, 28, 32, 36, 39, 46–48, 50, 58, 60, 63, 64, 68, 69]
	2019–2020	19 (38.8%)	[23, 27, 30, 33, 35, 37, 38, 41, 42, 51–55, 57, 59, 61, 62, 67]
	2017–2018	14 (28.6%)	[21, 22, 24, 29, 31, 34, 40, 43–45, 49, 56, 65, 66]
Objective^a^	Describe and discuss engagement activities	20 (40.8%)	[22–27, 29–31, 36, 37, 40, 41, 44, 45, 49, 50, 62, 63, 68]
	Examine researcher experiences	13 (26.5%)	[26, 33, 34, 38, 47, 48, 51, 56–61]
	Examine PWLE experiences	10 (20.4%)	[21, 23, 26, 32, 33, 39, 47, 53, 55, 69]
	Examine the impact of engagement	4 (8.2%)	[36, 64, 65, 68]
	Recommend engagement practices	4 (8.2%)	[31, 39, 43, 64]
	Other	10 (20.4%)	[27, 28, 35, 42, 46, 52, 54, 66, 67, 69]

*Note. PWLE* people with lived experience of mental health and/or substance use challenges

^a^Sum of percentages exceeds 100% because some articles had multiple objectives

**Table 5:** Summary of research evidence and practice gaps

Review question	Overarching gap	Components
Research evidence gaps	Conceptualize engagement	Define PWLE, standardize terminology, standardize accessible reporting
	Develop resources	Best practice guidelines, concrete tools, process descriptions to increase clarity
	Evaluate engagement	Impact on research, impact on PWLE, impact on researchers, using various methodologies, conducted with PWLE and considering best practices
	Increase diversity	Generate research including representative populations, evaluate impact among diverse PWLE
Implementation gaps	Broader institutional gaps	Sufficient and flexible funding; support of institutions, funders, ethics boards; community collaborations
	Day-to-day practice gaps	Clear, early planning; building relationships/rapport; training and mentorship; increased diversity; PWLE at leadership levels

*Note. PWLE* people with lived experience

## Research evidence gaps

Research teams identified several research evidence gaps to be addressed to improve research on PWLE engagement. Research evidence gaps included four categories: (1) conceptualizing engagement, (2) establishing resources, (3) increasing diversity, (4) evaluating engagement.

### Conceptualizing PWLE engagement

Research teams noted several gaps in the overarching conceptualization of PWLE engagement, highlighting the need to address these gaps in future research. Conceptualization gaps included the need to further reflect on what lived experience is and who is considered a PWLE [28, 30, 33]. For example, research teams raised issues related to the continuum of research knowledge among PWLE. This included different degrees of experience in engagement among PWLE who are new to engagement versus those who are experienced in these roles and among those without academic training, as well as the complex interplay in roles experienced by PWLE who also have academic training and experience [28, 33, 45, 49, 59, 66]. Further areas of conceptual clarification included the standardization of terminology and keywords describing PWLE engagement. Currently, these are varied, lacking in guidance, and not subject to consensus [53, 63]. Likewise, the standardization of reporting that is inclusive and accessible to non-researchers (e.g., jargon-free) was called for. It was noted that it can sometimes be unclear who the PWLE members are on a research team and how they contributed to a given project, making contributions and impacts difficult to determine [33, 37, 53, 60, 63, 68].

### Establishing resources

Authors of the selected studies highlighted that research work is needed to equip researchers with comprehensive resources that provide guidance on practical and methodological approaches to conducting PWLE engagement. This was inclusive of a range of types of resources, such as engagement practice recommendations, concrete tools, and publications that describe engagement processes to better support teams embarking on PWLE engagement [21, 31, 33, 40, 50, 51, 63, 65, 69]. A lack of clarity in the practices that should be followed when engaging PLWE in research, versus the practices that are actually followed, was highlighted as a major research evidence gap [22, 34, 51, 59, 60, 67, 69].

### Increasing diversity

Given the lack of diversity in engagement teams, increasing the diversity of PWLE engagement was both a research and implementation gap. As a research evidence gap, several authors highlighted the need to generate future research that includes PWLE from diverse and representative groups to expand the evidence related to both engagement experiences and the impact of engagement to diverse populations that are traditionally excluded from engagement [21, 25, 35, 37, 53, 61–63].

### Evaluating engagement

A notable research evidence gap consistently identified by authors conducting PWLE engagement in studies was the need to evaluate engagement. Research teams highlighted the importance of evaluating the impacts of engagement on research projects [28, 42, 53], as well as on PWLE and researchers [22, 27, 42, 53, 58], using a variety of methodologies [22, 25, 34, 56, 69]. Evaluations should be conducted with PWLE [51] while considering best practices in engagement [25].

## Implementation gaps

A wide range of gaps were identified concerning the implementation of PWLE engagement in research. Gaps were identified in two overarching categories: (1) broader institutional gaps, and (2) gaps in the day-to-day practice of PLWE engagement.

### Broader institutional gaps

Research teams identified a number of implementation gaps at the broader institutional level. These included gaps in funding, institutional support, and the understanding of engagement among research ethics boards. In terms of funding, research teams highlighted the need for more funding and more flexible funding mechanisms to support PWLE engagement across the research lifespan [22, 27, 28, 31, 33, 34, 37, 38, 40, 41, 47, 59, 68]. This includes funding for the coordination of PWLE activities [28]. Furthermore, funding needs to be flexible, allowing for PWLE innovation before and after major research funding is awarded [25, 28, 31]. Research teams emphasized the importance of increasing institutional support for PWLE engagement, including support of research institutions [33, 34, 42, 47, 51, 61] and funding institutions [42, 53], as well as providing support for and entering into collaboration with community organizations with expertise in engagement [21, 22, 38]. Numerous teams highlighted the need to increase the understanding that research ethics boards have of PWLE engagement in research to facilitate engagement [21, 31, 38, 57, 59].

### Day-to-day practice

A number of areas of gaps with regard to the day-to-day practice of implementing engagement were identified, namely: (1) clear, early planning of PWLE engagement; (2) building relationships and rapport; (3) providing appropriate training and mentorship; (4) increasing diversity; (5) embedding PWLE engagement in leadership.

Clearer planning for PWLE engagement was called for, in the form of the development of appropriate plans setting out the engagement activities and timelines [28, 36–39, 62, 67], which must occur as early as possible in the research process [22, 24, 27–29, 41, 45, 47, 62, 67]. Early engagement would include collecting PWLE perspectives at the stages of generating research questions, designing studies, and submitting funding applications. Clear, early plans outlining the ‘who,’ ‘what,’ ‘when,’ ‘where,’ and ‘why’ of PWLE engagement were seen as important to improving project development and funding applications, while improving response to tight deadlines and adding flexibility.

The importance of building strong relationships, good partnerships, and positive rapport with PWLE was highlighted as a need and an implementation gap [25, 30, 33, 36, 45, 47, 57, 58, 64, 67]. This included improving researcher attitudes toward engagement [45, 56, 57] and equalizing power dynamics throughout the research process [22, 25, 27, 30–34, 36, 45, 46, 57, 60, 65, 68, 69]. Using preferred language, avoiding research jargon, conducting debriefs, ensuring clear communication, and taking the time to develop rapport to create a safe space were some examples of mechanisms applied to build strong relationships, although more work in this area is required.

Many research teams highlighted an implementation gap in terms of the need for strong training. This gap was inclusive of training for PWLE that enables them to contribute authentically to research [22, 25, 51, 59, 65, 68], as well as training for researchers and research staff to enable them to work with PWLE authentically and prevent tokenistic engagement [42, 57, 58, 68]. Training gaps extended beyond initial training to also include networking, ongoing mentorship, and support for all groups [22, 31, 34, 38, 42, 59]. Types of training recommended included one-on-one training, workshops, and matching of PLWE with established researchers, in the form of initial training and ongoing professional development.

Gaps regarding diversity in the implementation of engagement were a key concern of some research teams [28, 33, 62, 69]. Authors highlighted gaps in PLWE representativeness of the target population in terms of lived experience (i.e., *lived experience of what?*) and demographic characteristics, which can lead to missing the voices of certain equity-deserving populations in informing research [30, 41, 45].

Some research teams further highlighted the lack of PWLE at the leadership level of research, which is a gap that could be addressed to strengthen engagement activities [21, 25, 28, 34, 58].

## Discussion

This scoping review synthesized articles about engaging PWLE in mental health and substance use research to understand the key research evidence and implementation gaps identified by research teams working in this manner. Identified research gaps included conceptualizing PLWE engagement, developing resources, conducting research with diverse PLWE teams, and evaluating the impacts of PWLE engagement. Implementation gaps were more varied and revolved around broader institutional gaps and day-to-day practice gaps. These results highlight many areas on which researchers and engagement teams should focus their efforts to guide and improve PLWE engagement in mental health and substance use research.

When reviewing the papers, it was clear that many authors described implementing an array of positive engagement practices. However, they did so in idiosyncratic ways, without anchoring their implementation of engagement to best practice guidelines or consistent reporting, making it difficult to understand or report on the way in which each team engaged PWLE in their work. The research evidence gaps pointed to the need to develop concrete resources and a need for standardization, while the implementation gaps pointed to several areas for positive practices in engagement where further progress is required. Standardizing methods for conducting PWLE engagement in research, along with the standardization of language and reporting, are potential future directions to improve the quality of engagement practices and consistency among research reports [4]. Transforming various implementation and engagement practice recommendations into standardized, rigorous practice guidelines that encompass enough flexibility to adapt to the local context [70] may support researchers in conducting authentic engagement and reporting on it clearly. Concrete guidelines might address the implementation gaps that can support day-to-day practice, such as *how to* build relationships, *how to* plan for engagement, and *how to* equalize power dynamics to set the stage for authentic engagement. Such recommendations could be supported by some of the broad-based resources for researchers and training materials from PWLE engagement organizations [71–73]. The use of reporting guidelines, such as the Guidance for Reporting Involvement of Patients and the Public (GRIPP2) reporting checklists [15], may be a future direction for more consistent reporting on engagement activities, for example guiding more consistent reporting on the ways in which PWLE have been engaged. However, any efforts to standardize engagement should be balanced against the need for flexibility [70, 74].

Strong institutional support is an established facilitator of PWLE engagement and can enhance engagement activities broadly [4, 13, 75]. Institutions and funders are encouraged to support PWLE engagement by valuing the contributions of PWLE and PWLE-engaged researchers, but also by taking concrete steps to support them, such as providing funding to enable them to begin engagement early, prior to the receipt of large funding awards, and by working with them to establish ethics review processes that support engagement. They might also consider reinforcing the standardized reporting of the engagement institutionally, using tools like the GRIPP2 checklist [15] or using other flexible reporting standards at the internal, funder, or ethics board reporting levels. Guidance from the implementation science literature may support the implementation of strong engagement practices within and across institutions [76].

Many research teams emphasized the need to evaluate engagement. Indeed, a more substantial empirical evidence based on the impacts of engagement may increase buy-in among researchers and institutions alike. However, the purpose and implications of evaluative work should be carefully considered. Notably, some have raised cautions about evaluating the impact of engagement from an ethical standpoint [77, 78]. They argue that by over-emphasizing the impacts of engagement on the research process and the resulting findings, researchers may obscure the ethical imperative of engaging PWLE in research as a means of democratizing research. It is important to note that engagement should continue to happen regardless of empirical evidence for effectiveness, i.e., null findings of impact should in no way attenuate calls to conduct research engaging PWLE. While research teams have consistently argued for a need to evaluate engagement’s impact on research and on the individual experiences of PLWE and researchers, caution should be exercised in over-emphasizing impacts on the research process as it may overshadow the ethical imperative for engagement.

Increasing diversity in engagement emerges as both a research and implementation gap, given gaps in diversity in engagement as a whole [79]. Inadequate diversity and representation can be associated with tokenistic engagement [80], making the implementation gaps in diversity a considerable concern. It is important to keep in mind that PWLE are not a single, homogeneous group, and the PWLE engaged should reflect characteristics of the population addressed by the research. If the PWLE engaged are not representative of the population addressed by the research, the engagement is not authentic and democratizing, as it is intended to be [81], and certain voices can overpower others. The development of equity-oriented engagement practices that explicitly include equity-deserving groups is important across health research [82]. Research teams are encouraged to consider how to expand their engagement initiatives to include more diverse, representative voices and to evaluate engagement through a diversity-focused and anti-oppressive lens.

This review has strengths and limitations to consider. We used qualitative codebook analysis to synthesize research evidence and implementation gaps in PWLE engagement as reported by research teams. We engaged PWLE in the review process to ensure relevance. To capture the current evidence gaps, this review covered only the past five years. Any gaps identified outside of that period would have been missed. Other research evidence and implementation gaps that have not been expressed by researchers in peer review manuscripts may exist. Furthermore, gaps experienced by PWLE and not reported on by research teams would not have been reported. Additional research approaches might be considered to develop a consensus on the most urgent priorities in this area of work from PWLE and researcher perspectives. It should also be noted that the articles reviewed were published by teams who are likely conducting engagement with at least some degree of success. The research and implementation gaps, therefore, emerge from a limited and biased sample of researchers who believe in engagement and are willing to work through its challenges [68]. Different evidence and implementation gaps may emerge from teams that are not engaging PWLE in their research. Understanding the additional gaps experienced outside of the PLWE engagement community is an area for future work. It should be noted that some literature may have been missed. A thorough search included keywords inclusive of mental health and substance use. However, mental health research is a primary focus of the team and the results are limited in terms of the engagement of people who use substances, potentially missing some substance-specific engagement gaps. Future research should specifically examine these questions in the substance use sphere. Since this review focused on academic research, non-academic, community-based engagement learnings would also have been missed and may have important findings to contribute to this sphere.

## Conclusions

Despite progress in PWLE engagement in mental health and substance use research in recent years, research evidence and implementation gaps remain. Continued research to understand how to conduct authentic engagement of diverse populations and evaluate its impact is required. Also required is further attention to the conceptualization, institutional commitment, and day-to-day practice of engagement, alongside the development of resources to support PWLE engagement. Research teams are encouraged to conduct ongoing PWLE-engaged research and research on the science of PLWE engagement to address these gaps in a rigorous manner.

## Data Availability

The datasets used and/or analysed during the current study are available from the corresponding author on reasonable request.

## Abbreviations

PWLE: People with lived experience
GRIPP2: Guidance for Reporting Involvement of Patients and the Public
PRISMA: Preferred Reporting Items for Systematic Reviews and Meta-Analyses
